# Fenofibrate Decreases Hepatic P-Glycoprotein in a Rat Model of Hereditary Hypertriglyceridemia

**DOI:** 10.3389/fphar.2019.00056

**Published:** 2019-02-07

**Authors:** Martin Poruba, Zuzana Matuskova, Martina Hüttl, Hana Malinska, Olena Oliyarnyk, Irena Markova, Sona Gurska, Ludmila Kazdova, Rostislav Vecera

**Affiliations:** ^1^ Department of Pharmacology, Faculty of Medicine and Dentistry, Palacky University Olomouc, Olomouc, Czechia; ^2^ Institute of Molecular and Translational Medicine, Faculty of Medicine and Dentistry, Palacky University Olomouc, Olomouc, Czechia; ^3^ Center of Experimental Medicine, Institute for Clinical and Experimental Medicine, Prague, Czechia

**Keywords:** fenofibrate, P-glycoprotein, Mdr1, drug-drug interactions, hypertriglyceridemia, metabolic syndrome

## Abstract

P-glycoprotein (P-gp) is a membrane-bound transporter encoded by Mdr1a/Abcb1a and Mdr1b/Abcb1b genes in rodents involved in the efflux of cytotoxic chemicals and metabolites from cells. Modulation of its activity influences P-gp-mediated drug delivery and drug-drug interaction (DDI). In the current study, we tested the effects of fenofibrate on P-gp mRNA and protein content in non-obese model of metabolic syndrome. Males hereditary hypertriglyceridemic rats (HHTg) were fed standard laboratory diet (STD) (Controls) supplemented with micronized fenofibrate in lower (25 mg/kg b. wt./day) or in higher (100 mg/kg b. wt./day) dose for 4 weeks. Liver was used for the subsequent mRNA and protein content analysis. Fenofibrate in lower dose decreased hepatic Mdr1a by 75% and Mdr1b by 85%, while fenofibrate in higher dose decreased Mdr1a by 90% and Mdr1b by 92%. P-gp protein content in the liver was decreased by 74% in rat treated with fenofibrate at lower dose and by 88% in rats using fenofibrate at higher dose. These findings demonstrate for the first time that fenofibrate decreases both mRNA and protein amount of P-gp and suggest that fenofibrate could affect bioavailability and interaction of drugs used to treat dyslipidemia-induced metabolic disorders.

## Introduction

P-glycoprotein (P-gp), encoded by the ABCB1 gene, is an ATP-dependent drug transporter localized mainly in an apical or luminal cell membrane. P-gp is responsible for the efflux of a wide range of structurally diverse metabolites and cytotoxic chemicals from cells and may play a role in drug-drug interactions (DDI) (Glaeser, 2011). In humans, P-gp is a 170-kDa membrane protein encoded by the Mdr1/Abcb1 gene (Ueda et al., 1987). In rodents, there are two genes Mdr1a/Abcb1a and Mdr1b/Abcb1b encoding P-gp enzyme (Devault and Gros, 1990).

Recent studies *in vitro* demonstrated that P-gp function is associated with its lipid membrane bilayer, which modulates substrate interaction, ATP hydrolysis, and drug transportation, and can influence DDI (Sharom, 2014).

Fenofibrate is a lipid-lowering drug used to treat hypertriglyceridemia and is often used in combination with other medications in patients with metabolic syndrome and cardiovascular complications as atrial fibrillation with the risk of stroke or patients with combined hyperlipidemia. Typical P-gp substrates are digoxin, rivaroxaban, dabigatran, or loperamide (Montesinos et al., 2014; Vranckx et al., 2018). Ehrhardt et al. (2004) demonstrated that fenofibrate inhibits P-gp *in vitro* with potency like simvastatin. So far, there are no data about possible P-gp-modulating effects of fenofibrate *in vivo* in chronically elevated hypertriglyceridemia.

In this preliminary study, we tested the effects of fenofibrate on the P-gp mRNA and protein level in rats *in vivo*. We used non-obese hereditary hypertriglyceridemic rats (HHTg) as a unique model for studying metabolic syndrome-related disorders such as hypertriglyceridemia, liver steatosis, impaired glucose tolerance, and insulin resistance (Vrana and Kazdova, 1990; Zicha et al., 2006). These metabolic disorders increase the risk of comorbidity and polypharmacy.

## Materials and Methods

### Animals

For the study, the hereditary hypertriglyceridemic (HHTg) rats were obtained from the Institute for the Clinical and Experimental Medicine (Prague, Czech Republic). Animals were kept in temperature controlled conditions under a 12:12 h light-dark cycle. All experiments were performed in agreement with the Animal Protection Law of the Czech Republic Act. No 359/2012 Coll. All procedures with animals were approved by the Ethics Committee, Ministry of Youth and Sports, Czech Republic.

### Study Design and Sampling

Male HHTg rats (4 months old) were fed standard laboratory diet (STD, Control; *n* = 6), or STD supplemented with micronized fenofibrate in lower dose (25 mg/kg b. wt./day; *n* = 6), or in higher dose (100 mg/kg b. wt./day; *n* = 6) for 4 weeks. Doses of fenofibrate were chosen according to the literature (Ibarra-Lara et al., 2017).

Micronized fenofibrate (Fenofix^®^ 200 mg cps) was purchased from Ingers Industrial Solutions, Czech Republic. At the end of the experiment, animals were sacrificed, and the livers were collected for the subsequent analysis.

### Gene Expression Assay

For the isolation of total mRNA, the RNeasy Plus Mini Kit (Qiagen, Valencia, CA, USA) was used, and thereafter, 1 μg of isolated RNA was used for the synthesis of cDNA using a Transcriptor High Fidelity cDNA synthesis kit (Roche Diagnostics, GmbH, Mannheim, Germany). The analysis was performed in LightCycler 1536 Instrument (Roche), and obtained data were normalized using the ^ΔΔ^Ct method. Used Taqman probes combining primer and probe with FAM or VIC dye and quencher (Abcb1a, Abcb1b, and Hprt1) were purchased from Life Technologies (Carlsbad, CA, USA). For the calculations of relative fold change, the “Delta-Delta Ct method” was used, and each sample was simultaneously normalized to internal control Hprt mRNA present (Livak and Schmittgen, 2001).

### Western Blotting

Homogenate samples containing 30 μg of lysed protein were electrophoresed on a Mini-PROTEAN^®^ TGX^TM^ gel (Bio-Rad Laboratories, Hercules, CA, USA), transferred onto a PVDF membrane (Trans-Blot^®^ Turbo^TM^ Midi PVDF Transfer Packs, Bio-Rad Laboratories, Hercules, CA, USA) using Trans-Blot^®^ Turbo^TM^ Transfer System (Bio-Rad Laboratories, Hercules, CA, USA), and blocked in milk for 1 h, incubated with primary monoclonal antibodies (ABCB1, β-tubulin, Cell Signaling Technology, Danvers, MA, USA) overnight at 4°C. Membranes were then washed and incubated with the corresponding secondary antibody (Sigma-Aldrich, St. Louis, MO, USA). Proteins were detected using Luminol reagent (WB, Luminol reagent, Santa Cruz, CA, USA) and medical X-ray films (Agfa, Belgium). For the protein quantification, CanoScan Toolbox software, ver. 5.0 (Canon Europa, Amstelveen, the Netherlands) and ElfoMan software, ver. 2.6 (Semecky Inc., Prague, Czech Republic) were used.

### Statistical Analysis

The normal distribution of data was tested using Shapiro-Wilk test. Statistical analysis was performed by ANOVA and subsequent *post hoc* Bonferroni test using Statistica software (ver. 12, Statsoft CZ, Prague, Czech Republic). Statistical significance was defined as *p* < 0.05.

## Results

**Figure 1:**
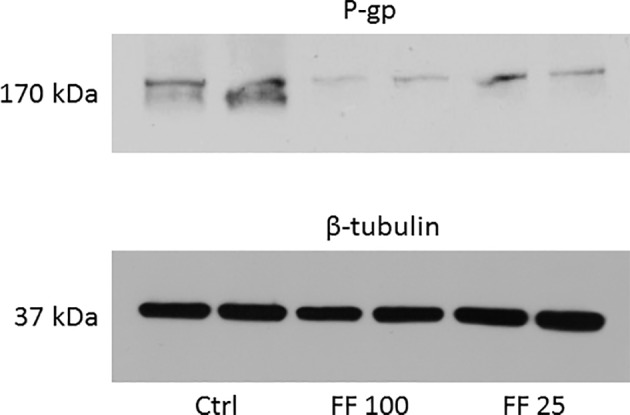
Representative Western blot of P-gp protein. Data are shown in duplicates in columns. Ctrl, control; FF100, fenofibrate 100 mg/kg b. wt/day; FF25, fenofibrate 25 mg/kg b. wt/day.

As shown in Table 1, ABCB1 protein content (P-gp) was decreased by 74% in rat treated with fenofibrate at a dose of 25 mg/kg b. wt. and by 88% in rats using fenofibrate at a dose of 100 mg/kg b. wt. Representative image of Western blot analysis of P-gp protein is shown in Figure 1. Our results show a highly significant decrease expression of mRNA of two monitored genes. Fenofibrate at a dose of 25 mg/kg b. wt. significantly decreased Mdr1a by 75% and Mdr1b by 85% (*p* < 0.0001 both). Fenofibrate in higher dose 100 mg/kg b.w. significantly decreased Mdr1a by 90% and Mdr1b by 92% (*p* < 0.0001 for both). Results are summarized in Table 1.

**Table 1:** Hepatic mRNA and protein content of P-glycoprotein (P-gp) in rats treated without or with fenofibrate at a dose of 25 or 100 mg/kg b. wt.

	Control	*p*^1^	Fenofibrate 25 mg/kg b.wt.	*p*^2^	Fenofibrate 100 mg/kg b.wt.
P-Glycoprotein	1.00 ± 0.09	<0.0001	0.26 ± 0.10	<0.0001	0.12 ± 0.04
Mdr1a/Abcb1a	1.00 ± 0.12	<0.0001	0.25 ± 0.09	<0.0001	0.10 ± 0.02
Mdr1b/Abcb1b	1.00 ± 0.15	<0.0001	0.15 ± 0.02	<0.0001	0.08 ± 0.01

Data are expressed as mean ± SD. *p* values denote differences:

1 p: Control vs. fenofibrate 25 mg/kg b. wt/day.

2 p: Control vs. fenofibrate 100 mg/kg b. wt/day.

## Discussion

The P-gp is expressed in the plasma membrane of cells in organs with barrier and elimination function where it plays an important role in the efflux of different drugs and xenobiotic from the cells as well as in the drug resistance development. In the current study, we proved an effect of fenofibrate on P-gp level in rats. We found out for the first time that hepatic mRNA of both Abcb1a/Mdr1a and Abcb1b/Mdr1b genes, as well as protein content of ABCB1, was significantly decreased in fenofibrate-treated hypertriglyceridemic rats with moderate hepatic steatosis. Our results are consistent with the experiments conducted on cell lines. Ehrhardt et al. (2004) showed that an inhibition of P-gp in the cell line with overexpression of human P-gp caused by fenofibrate was similar to simvastatin. The similar study by Yamazaki et al. (2005) showed a moderate inhibition of P-gp by fenofibrate measured in a P-gp overexpressing cell line according to the cellular accumulation of vinblastine. Moreover, our results showed a slight dose-dependent decrease in mRNA and protein content of studied transporter even though they did not achieve statistically significant differences between used doses. This suggests that this effect could be more pronounced in case of fenofibrate overdose or when used concurrently with another potent P-gp inhibitor. Interaction of fibrates with statins is often used in patients with dyslipidemia at high risk for cardiovascular diseases. Mild transiently increased levels of serum aminotransferases and liver injury were reported (Geng et al., 2013). Previously, we showed that combination of fibrate with rosuvastatin can induce liver damage in rats expressing the human C-reactive protein (Silhavy et al., 2015). P-gp in the liver plays an important role in the first-pass effect and limits drugs bioavailability. There are many known substrates, inhibitors, or inducers of P-gp, with a significant drug-drug interaction potential such as digoxin, amiodarone, or verapamil, which are frequently used to treat dyslipidemia-induced metabolic disorders (Schinkel et al., 1995). Our findings suggest that fenofibrate could increase a bioavailability of concomitantly administered drugs by reducing their liver excretion. On the other hand, different studies showing that another PPARα inducer – clofibrate – increased P-gp expression independent of PPARα induction suggest a possible organ and animal specificity in P-gp activity (Kok et al., 2003; More et al., 2017). Further studies are needed to confirm these effects.

Although P-gp is generally considered as a membrane efflux transporter of drugs and xenobiotic, its role is more complex and plays a role in the transport of some endogenous compounds like lipids (Aye et al., 2009). It was found that mice lacking P-gp accumulate higher plasma and tissue concentrations of P-gp substrates such as glucocorticoids and may develop hepatic steatosis and obesity (Foucaud-Vignault et al., 2011). Fenofibrate as a lipid-lowering drug could ameliorate some of these negative effects. Further studies are needed to clarify the role of P-gp in lipid homeostasis and in membrane lipid bilayer composition which affects drug binding to P-gp.

In conclusion, our preliminary result demonstrated that fenofibrate treatment and especially the overdose of rats with chronic hypertriglyceridemia and mild hepatic steatosis decreased mRNA of Abcb1a/Mdr1a and Abcb1b/Mdr1b genes as well as the protein content of ABCB1. These results suggest that fenofibrate could affect bioavailability and interaction of drugs used to treat dyslipidemia-induced metabolic disorders. The limitation of our study is that P-gp content was determined only in the liver not in other tissues such as intestine or blood-brain barrier. To elucidate the role of P-gp in the mechanism of metabolic effects of fenofibrate and other drugs in the treatment of dyslipidemia, further studies are needed, including studies of P-gp content in the liver and other important tissues.

## Author Contributions

MP, ZM, MH, HM, OO, IM, and SG conceptualized and performed the experiments. MP and ZM analyzed results. MP with the help and supervision of LK and RV wrote the paper.

### Conflict of Interest Statement

The authors declare that the research was conducted in the absence of any commercial or financial relationships that could be construed as a potential conflict of interest.
